# Ticks and rickettsiae from wildlife in Belize, Central America

**DOI:** 10.1186/s13071-016-1348-1

**Published:** 2016-02-02

**Authors:** Marcos G. Lopes, Joares May Junior, Rebecca J Foster, Bart J Harmsen, Emma Sanchez, Thiago F. Martins, Howard Quigley, Arlei Marcili, Marcelo B. Labruna

**Affiliations:** Department of Preventive Veterinary medicine and animal Health, Faculty of Veterinary Medicine, University of São Paulo, São Paulo, SP 05508-270 Brazil; Panthera, 8 West, 40th street, 18th Floor, New York, NY 10018 USA; Universidade do Sul de Santa Catarina, Av. José Acácio Moreira, 787, Bairro Dehon, Caixa Postal 370, CEP 88704-900 Tubarão, SC Brazil; Environmental Research Institute, University of Belize, UB preschool Grounds, Price Centre Road, PO box 340, Belmopan, Belize; Universidade de Santo Amaro, R. Prof. Enéas de Siqueira Neto, 340, CEP 04829-300 São Paulo, SP Brazil

**Keywords:** *Amblyomma ovale*, *Amblyomma pacae*, *Amblyomma oblongoguttatum*, *Ixodes affinis*, *Rickettsia*, Wild animals, Central America

## Abstract

**Background:**

The agents of spotted fevers in Latin America are *Rickettsia rickettsii*, *R. parkeri*, *Rickettsia* sp. strain Atlantic rainforest, and *R. massiliae*. In Continental Central America, *R. rickettsii* remains the only known pathogenic tick-borne rickettsia. In the present study, ticks were collected from wild mammals in natural areas of Belize. Besides providing new data of ticks from Belize, we investigated rickettsial infection in some of these ticks. Our results provide ticks harboring rickettsial agents for the first time in Central America.

**Methods:**

Between 2010 and 2015, wild mammals were lived-trapped in the tropical broadleaf moist forests of central and southern Belize. Ticks were collected from the animals and identified to species by morphological and molecular analysis (DNA sequence of the tick mitochondrial 16S RNA gene). Some of the ticks were tested for rickettsial infection by molecular methods (DNA sequences of the rickettsial *gltA* and *ompA* genes).

**Results:**

A total of 84 ticks were collected from 8 individual hosts, as follows: *Amblyomma pacae* from 3 *Cuniculus paca*; *Amblyomma ovale* and *Amblyomma coelebs* from a *Nasua narica*; *A. ovale* from an *Eira Barbara*; *A. ovale*, *Amblyomma* cf. *oblongoguttatum*, and *Ixodes affinis* from a *Puma concolor*; and *A. ovale*, *A. coelebs, A*. cf. *oblongoguttatum*, and *I. affinis* from two *Panthera onca*. Three rickettsial agents were detected: *Rickettsia amblyommii* in *A. pacae, Rickettsia* sp. strain Atlantic rainforest in *A. ovale,* and *Rickettsia* sp. endosymbiont in *Ixodes affinis*.

**Conclusions:**

The present study provides unprecedented records of ticks harboring rickettsial agents in the New World. An emerging rickettsial pathogen of South America, *Rickettsia* sp. strain Atlantic rainforest, is reported for the first time in Central America. Besides expanding the distribution of 3 rickettsial agents in Central America, our results highlight the possible occurrence of *Rickettsia* sp. strain Atlantic rainforest-caused spotted fever human cases in Belize, since its possible vector, *A. ovale*, is recognized as one of the most important human-biting ticks in the Neotropical region.

## Background

Belize is a tropical country occupying an area of only 22,800 km^2^, <5 % of continental Central America. Despite its relatively small land, Belize is recognized by its high biodiversity, especially wildlife. Among 900 tick species of the world, nearly 200 are found in the Neotropical Zoogeographic region [1, 2]. The tick fauna of Belize is currently composed of 18 species, distributed in the genera *Amblyomma* (13 species), *Ixodes* (2), *Rhipicephalus* (2), and *Dermacentor* (1) [1, 3].

Tick-borne rickettsiae are bacterial agents of the genus *Rickettsia* that cause emerging and re-emerging zoonoses worldwide [4]. Recent reviews on the occurrence of *Rickettsia* species in Latin America indicated that only 3 tick-borne *Rickettsia* species occur in Central American main land, in contrast to at least 9 rickettsial agents infecting ticks in South America [4, 5]. In fact, until 2009, *Rickettsia rickettsii,* the agent of Rocky Mountain spotted fever, was the only tick-borne rickettsia known to occur in Central America, with reports from Panama and Costa Rica [5]. In these two countries, *R. rickettsii* has been associated with three tick species, *Amblyomma mixtum* (reported as *Amblyomma cajennense*), *Dermacentor nitens,* and *Haemaphysalis leporispalustris* [6–8]. Since 2009, two more tick-borne *Rickettsia* species have been reported in continental Central America: *Rickettsia bellii* infecting *Amblyomma sabanerae* from El Salvador and Costa Rica [9, 10] and *Amblyomma rotundatum* from Panama [11]; and *Rickettsia amblyommii* infecting *A. mixtum* (reported as *A. cajennense*) from Panama, Costa Rica, and Honduras [8, 12, 13] and *Amblyomma longirostre* from Costa Rica and Honduras [10, 13]. Additionally, at least three uncultured rickettsial agents have been reported in Central America, namely *Rickettsia* sp. strain IbR-CRC in *Ixodes boliviensis* from Costa Rica [14], *Rickettsia* sp. endosymbiont (strain Barva) in *Ixodes minor* from Costa Rica [10], and *Rickettsia* sp. strain Colombianensi in *Amblyomma dissimile* from Honduras [13].

The current scenario of tick-borne rickettsiae in Latin America is still modest, compared to Europe, where more than 20 tick-borne agents have been reported [4]. Moreover, whereas nearly 10 tick-borne rickettsiae are agents of human illness (generally known as spotted fevers) in Europe, only 4 tick-borne rickettsiae are known to cause spotted fevers in humans in Latin America [4]. The agents of spotted fevers in Latin America are *R. rickettsii, R. parkeri, Rickettsia* sp. strain Atlantic rainforest, and *R. massiliae* [4]. In Continental Central America, *R. rickettsii* has remained as the only known pathogenic tick-borne rickettsia.

In the present study, ticks were collected from wild mammals in natural areas of Belize. Besides providing new data of ticks from Belize, we investigated rickettsial infection in some of these ticks. Our results provide ticks harboring rickettsial agents for the first time in Central America.

## Methods

Between 2010 and 2015, wild mammals were lived-trapped in the tropical broadleaf moist forests of central and southern Belize, as part of a study of wildlife movement. Ticks were collected from the animals and stored in plastic tubes containing 92 % ethanol. In the laboratory, ticks were identified to species through morphological examination following specific identification keys for *Amblyomma* species [15, 16], and for *Ixodes* species [17, 18]. Morphological identification was confirmed by molecular analysis. For this purpose, representative specimens of each tick species were submitted to DNA extraction using the guanidine isothiocyanate phenol technique [19] and tested by a PCR assay targeting a portion of the tick mitochondrial 16S rRNA gene, as previously described [20]. Amplicons were purified with ExoSap (USB, Cleveland, Ohio, USA) and DNA-sequenced in an ABI automated sequencer (Applied Biosystems/Thermo Fisher Scientific, model ABI 3500 Genetic Analyzer, Foster City, California, USA) with the same primers used for PCR. The sequences obtained were submitted to blast analyses (www.ncbi.nlm.nih.gov/blast) to infer the closest similarities available in GenBank.

The mitochondrial 16S rRNA partial sequences of *Ixodes* ticks collected in the present study were aligned with corresponding 16S rRNA sequences of different *Ixodes* species available in GenBank, which included mostly those species belonging to the *Ixodes ricinus* species complex, as recently reported [21]. The sequence of *Ixodes uriae* was used as outgroup. Phylogenetic trees were inferred by the maximum parsimony methods and were performed with PAUP 4.0b10 software [22] with 1000 replicates of random-addition taxa and tree bisection and reconnection branch swapping; all positions were equally weighed.

Tick DNA samples were screened for rickettsial infection by a PCR protocol using primers CS-78 and CS-323, targeting a 401-bp fragment of the rickettsial citrate synthase gene (*glt*A) [23], which is relatively conserved among *Rickettsia* species [24]. Samples yielding visible PCR products by this PCR were further tested with primers Rr190.70p and Rr190.701n, targeting a 631-bp fragment of the rickettsial 190-kDa outer membrane protein gene (*omp*A) [25]. In each set of reactions, negative control tubes containing water and a positive control tube containing DNA of *Rickettsia* sp. strain NOD were included. Amplicons were purified and DNA-sequenced as described above, and then submitted to blast analyses.

Ethical approval. This work was authorized by the Government of Belize’s Forest Department; research permits are CD/60/3/09(18), CD/60/3/10(44), CD/60/3/12(21), CD/60/3/15.

## Results

A total of 84 ticks were collected from 8 individual hosts, comprising 5 different tick species from 5 host species (Table 1). Four of the five tick species belonged to the genus *Amblyomma*, and were morphologically identified as *Amblyomma ovale, Amblyomma coelebs, Amblyomma pacae,* and *Amblyomma* cf. *oblongoguttatum.* This later species is provisionally treated with “cf.” because its external morphology presented slight differences to *A. oblongoguttatum* from Brazil (type locality of *A. oblongoguttatum*), which will be presented in detail in another manuscript, still in progress (M.B.L., unpublished data).

**Table 1:** Ticks (M: males, F: females, N: nymph, L: larva) collected from wild hosts in Belize and the rickettsial detection in part of the ticks

Vertebrate hosts				Tick species: No. per stage	Rickettsial detection in ticks	
No.	Order: Family	Species	Locality^a^		No. positive/No. tested (%)	*Rickettsia* species
1	Rodentia: Cuniculidae	*Cuniculus paca*	A	*Amblyomma pacae*: 1 M		
2		*C. paca*	A	*A. pacae*: 1 M		
3		*C. paca*	A	*A. pacae*: 2 M, 1 F	1/1 (100)	*R. amblyommii*
4	Carnivora: Procyonidae	*Nasua narica*	A	*Amblyomma ovale*: 1 M, 1 F	0/1 (0)	
				*Amblyomma coelebs*: 1 N		
5	Carnivora: Mustelidae	*Eira barbara*	A	*A. ovale*: 2 F	0/1 (0)	
6	Carnivora: Felidae	*Puma concolor*	B	*A. ovale*: 6 M	2/4 (50)	*Rickettsia* sp.^b^
				*Amblyomma* cf. *oblongoguttatum*: 1 M, 6 F	0/3 (0)	
				*Ixodes affinis*: 4 M, 6 F	4/4 (100)	*Rickettsia* sp.^c^
7		*Panthera onca*	B	*A. ovale*: 6 M	1/3 (33.3)	*Rickettsia* sp.^b^
				*A. coelebs*: 1 L	0/1 (0)	
				*A.* cf. *oblongoguttatum*: 3 M, 2 F, 1 L	0/2 (0)	
				*Ixodes affinis*: 3 M, 12 F	6/7 (85.7)	*Rickettsia* sp.^c^
8		*Panthera onca*	B	*A. ovale*: 2 M		
				*A.* cf. *oblongoguttatum*: 2 F		
				*A. coelebs*: 1 N		
				*Ixodes affinis*: 5 M, 13 F	8/9 (88.9)	*Rickettsia* sp.^c^

^a^A: Private lands, Cayo district; B: Cockscomb Basin Wildlife Sanctuary, Stann Creek district

^b^strain Atlantic rainforest

^c^rickettsia endosymbiont of *Ixodes affinis*

**Fig. 1:**
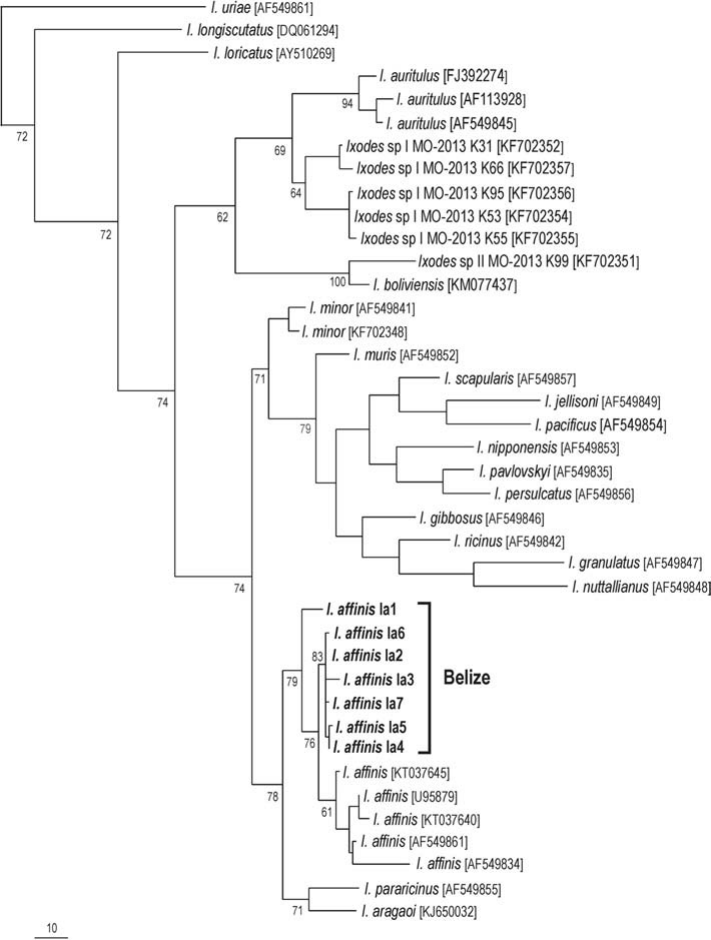
Maximum parsimony (MP) phylogenetic tree of 16S rDNA partial sequences of *Ixodes affinis* from Belize and other tick species of the genus *Ixodes*. The *Ixodes uriae* corresponding sequence was used as outgroup. Numbers at nodes are support values derived from bootstrap (1000 replicates). Numbers in brackets are GenBank accession numbers

Morphological identifications were corroborated by molecular analysis, as the mitochondrial 16S rRNA gene partial sequences of *A. ovale, A. coelebs,* and *A. pacae* matched ≥99 % similarity to conspecific sequences available in GenBank, while the sequences of *A.* cf. *oblongoguttatum* matched closest (≈92 % similarity) to a sequence of *A. oblongoguttatum* from Brazil (Table 2). Two *Amblyomma* larvae from the same individual host were molecularly identified as *A. coelebs* and *A.* cf. *oblongoguttatum* (Tables 1, 2). A total of 43 tick specimens belonged to the genus *Ixodes,* and were morphologically identified as *Ixodes affinis*. Through molecular analyses, the 16S rRNA partial sequences of 18 *Ixodes* specimens generated 7 different haplotypes, which were 97–99 % closest to corresponding sequences of *I. affinis* from southeastern United States (Table 2). In the phylogenetic analysis (Fig. 1), the 7 haplotypes of *I. affinis* from Belize formed a clade with 5 haplotypes of *I. affinis* from the United States [GenBank: U95879, AF549834, KT037645, KT037645] and Colombia [GenBank: AF549861], under moderate bootstrap support (79 %).

**Table 2:** Ticks processed by molecular analysis for determination of their 16S rRNA gene partial sequences, and their closest similarities in GenBank

Tick species	Specimens^a^	Haplotype code	Host No. in Table 1	Closest similarity in GenBank for the 16S rRNA gene
*Amblyomma ovale*	4 M, 1 F	Ao1	4,6,7	99.5 % *A. ovale* Brazil (KR605467)
	1 M	Ao2	6	99.0 % *A. ovale* Brazil (KR605467)
	1 F	Ao3	5	99.3 % *A. ovale* Brazil (KR605467)
	1 M	Ao4	7	99.3 % *A. ovale* Brazil (KR605467)
*A. pacae*	1 M	Ap1	3	99.7 % *A. pacae* Brazil (JX141384)
*A. coelebs*	1 L	Ac1	7	99.0 % *A. coelebs* Argentina (KM519936)
*A.* cf. *oblongoguttatum*	2 F,1 L	Acfo1	6,7	92.2 % *A. oblongoguttatum* Brazil (FJ424407)
	1 F	Acfo2	6	91.9 % *A. oblongoguttatum* Brazil (FJ424407)
*Ixodes affinis*	1 M	Ia1	7	97.3 % *I. affinis* USA (KT037643)
	1 M,9 F	Ia2	6,7,8	99.8 % *I. affinis* USA (KT037645)
	2 F	Ia3	7,8	98.8 % *I. affinis* USA (KT037648)
	1 M	Ia4	8	99.3 % *I. affinis* USA (KT037645)
	1 F	Ia5	8	99.3 % *I. affinis* USA (KT037645)
	1 F	Ia6	8	99.5 % *I. affinis* USA (KT037645)
	2 M	1a7	8	99.5 % *I. affinis* USA (KT037645)

^a^M male, F female, N nymph, L larva

A total of 36 tick specimens were tested for rickettsiae through PCR. Three rickettsial agents were detected: *Rickettsia amblyommii* in *A. pacae, Rickettsia* sp. strain Atlantic rainforest in *A. ovale,* and *Rickettsia* sp. endosymbiont of *Ixodes affinis* (Table 1). Both the *glt*A and the *omp*A partial sequences of *R. amblyommii* were 100 % identical to corresponding sequences in GenBank [JF694089, CP012420, respectively]. Both the *glt*A and the *omp*A partial sequences of *Rickettsia* sp. strain Atlantic rainforest were 100 % identical to corresponding sequences in GenBank [GQ855235, KM116015, respectively].

The *glt*A partial sequence of *Rickettsia* sp. endosymbiont of *Ixodes affinis* was 99.7 % (336/337-bp) identical to *Rickettsia* sp. IbR-CRC [GenBank: KJ507211], previously detected in *I. boliviensis* from Costa Rica, and 99.4 % (348/350-bp) identical to both *Rickettsia monancensis*, previously detected in *Ixodes ricinus* from Europe [GenBank: LN794217], and *Rickettsia* sp. Barva, previously detected in *I. minor* from Costa Rica [GenBank: KF702332]. The *ompA* partial sequence of *Rickettsia* sp. endosymbiont of *Ixodes affinis* was 99.8 % (586/587-bp) identical to *Rickettsia* sp. Barva, previously detected in *I. minor* from Costa Rica [GenBank: KF702334], 99.6 % (503/505-bp) identical to *Rickettsia* sp. IbR-CRC, previously detected in *I. boliviensis* from Costa Rica [GenBank: KJ507218], and 98.9 % (556/562-bp) and 99.0 % (536/541-bp) identical to the rickettsial endosymbionts of *Ixodes scapularis* [GenBank: XM002401667] and *Ixodes pacificus* [GenBank: GU047354], respectively.

Voucher tick specimens collected in the present study have been deposited at the tick collection “Coleção Nacional de Carrapatos” (CNC) under the accession numbers CNC-3159 to CNC-3166. GenBank nucleotide sequence accession numbers for the partial sequences generated in the present study are KU001155 - KU001169 for the 16S rRNA gene of *A. ovale, A. pacae, A. coelebs, A.* cf. *oblongoguttatum,* and *I. affinis*; KU001170, KU001173 for the *glt*A and *ompA* genes of *R. amblyommii;* KU001171, KU001174 for the *glt*A and *omp*A genes of *Rickettsia* sp. strain Atlantic rainforest; and KU001172, KU001175 for the *glt*A and *omp*A genes of *Rickettsia* endosymbiont of *I. affinis*.

## Discussion

The tick species found in the present study have been previously reported in Belize [26]. Our results are in agreement with previous studies, which indicated that *A. pacae* is a natural parasite of *C. paca* [27], and that *A. ovale* and *I. affinis* are natural parasites of Carnivora hosts in the Neotropical region [28, 29]. *Amblyomma coelebs* is primarily associated with tapirs, *Tapirus* spp. [30]. In fact, *Tapirus bairdii* is known to occur in the area where this tick was collected in the present study (data not shown). While the parasitism of immature stages of *A. coelebs* on *P. onca* has been previously reported [28], we provide the first report of *A. coelebs* nymph on *N. narica.* Furthermore, our findings of *A.* cf. *oblongoguttatum* on *P. concolor* and *P. onca* are in agreement with previous reports for *A. oblongoguttatum* infesting wild felids in Central America [29], and South America [27, 28]. However, at this moment, we are not confident that the taxon *A. oblongoguttatum* represents the same tick species in these two continents, based on slightly morphological differences (data not shown) and molecular analyses (Table 2). Because the type locality of *A. oblongoguttatum* is in Brazil [31], we are referring to the Belizean specimens as *A.* cf. *oblongoguttatum.*

Three (33.3 %) out of 9 *A. ovale* ticks were found to harbor the pathogen *Rickettsia* sp. strain Atlantic rainforest. This rickettsial agent was recently demonstrated to cause spotted fever in humans in Brazil [32, 33], where *A. ovale* was incriminated as a possible vector [34]. Until the present study, the occurrence of *Rickettsia* sp. strain Atlantic rainforest was restricted to Brazil, Colombia, and Argentina [4, 35, 36]. Thus, our results expand the distribution of this pathogen to Central America, where it must be considered a potential agent of human spotted fever. While previous studies showed that *Rickettsia* sp. strain Atlantic rainforest is phylogenetically related to *R. parkeri, R. africae,* and *R. sibirica* [32, 33, 35]*,* the definitive taxonomic status of this pathogen still needs to be established.

We provide the first record of *R. amblyommii* in the tick *A. pacae.* This rickettsial agent has a broad distribution in the New World, infecting a variety of *Amblyomma* species in the United States, Honduras, Costa Rica, Panama, French Guyana, Paraguay, Argentina, and Brazil [8, 10, 12, 13, 23, 37–40]. Currently, *R. amblyommii* is considered to be a potential human pathogen, since there have been serological evidence of human infection by this agent in the United States [41, 42].

Most of the *I. affinis* ticks of the present study were shown to harbor a rickettsial agent*.* This agent was considered to be an endosymbiont, based on previous studies on closely related *Ixodes* species (e.g., *I. scapularis, I. pacificus, I. ricinus, I. boliviensis*), which were also infected by genetically close-related rickettsial endosymbionts, usually under high infection rates [14, 43]. Further studies on isolation and deeper molecular characterization are needed to elucidate the taxonomic status of the rickettsial endosymbionts of *Ixodes* species from Latin America.

## Conclusions

The present study provides unprecedented records of ticks harboring rickettsial agents in the New World. An emerging rickettsial pathogen in South America, *Rickettsia* sp. strain Atlantic rainforest, is reported for the first time in Central America. Besides expanding the distribution of 3 rickettsial agents in Central America, our results highlight the possible occurrence of *Rickettsia* sp. strain Atlantic rainforest-caused spotted fever human cases in Belize, since its possible vector, *A. ovale,* is recognized as one of the most important human-biting ticks in the Neotropical region [27, 44].
